# Assessment of spectral ghost artifacts in echo-planar spectroscopic micro-imaging with flyback readout

**DOI:** 10.1038/s41598-024-73391-y

**Published:** 2024-09-24

**Authors:** Jan Weis, Magor Babos, Sergio Estrada, Ram Kumar Selvaraju

**Affiliations:** 1 Department of Medical Physics, Uppsala University Hospital, Uppsala, Sweden; 2 Mediso Medical Imaging Systems, Budapest, Hungary; 3 Department of Medicinal Chemistry, Uppsala University, Uppsala, Sweden

**Keywords:** Magnetic resonance, Echo-planar spectroscopic imaging, Flyback readout, Spectral ghost artifacts, High-spatial resolution, Micro-imaging, Biophysics, Health care, Medical research, Physics

## Abstract

In this work, echo-planar spectroscopic imaging (EPSI) with flyback readout gradient-echo train was implemented in a preclinical MR scanner. The aim of this study is to visualize and quantify the ghost spectral lines produced by two, three and four interleaved echo trains with different amplitudes of the readout gradients, and to investigate the feasibility of the flyback data acquisition in micro-imaging of small animals. Applied multi-slice EPSI sequence utilizes asymmetric gradient-echo train that combines the shortest possible rewind gradients with readout gradients. It simplifies data processing because all echoes are acquired with the same polarity of the readout gradient. The approach with four interleaved gradient-echo trains and with four echoes in each train provides broad spectral bandwidth in combination with narrow receiver bandwidth and a good water-fat signal separation. It improves signal-to-noise ratio without the undesired consequence of water-fat shift artifacts that are eliminated during data processing. Position, number, and intensity of the ghost spectral lines can be controlled by the suitable choice of spectral bandwidth, number of echo train interleaves, and the number of echoes in each interleave. This study demonstrates that high-spatial resolution EPSI with interleaved flyback readout gradient-echo trains is feasible on standard preclinical scanners.

## Introduction

Conventional proton (^1^H) magnetic resonance spectroscopic imaging (MRSI) phase-encodes two- or three spatial coordinates into the free induction decay (FID). This method is very time consuming when used at higher spatial resolution. To reduce acquisition time, echo-planar spectroscopic imaging (EPSI) was introduced^1,2^. The common feature of EPSI is the use of oscillating readout gradients which encodes one spatial and the spectroscopic dimension simultaneously in a single excitation. A majority of the EPSI sequences utilize gradient echo train with symmetrical trapezoidal positive and negative gradient pulses. The spectral bandwidth (sBW) is defined by the inter-echo time ΔTE (sBW = 1/ΔTE) of successive echoes used in the reconstruction of the spectra which in turn depends on the gradient hardware performance parameters such as gradient strength and slew rate. If sBW is too narrow due to gradient hardware limitations, the effective ΔTE may be decreased by multiple interleaved gradient echo trains^3–6^. The main drawback of EPSI is the fact that readout bandwidth (rBW), instead of sBW, is the decisive factor for signal-to-noise ratio (SNR). This leads to loss in SNR compared to conventional MRSI because rBW is larger than sBW. For the identical spectral matrix, voxel size, sBW, spectral resolution, and measurement time, the SNR of EPSI spectra is worse than spectra of conventional MRSI^7–9^. However, the speed of EPSI can be utilized in applications where SNR is not a limiting issue. Such an application is water-fat spectroscopy^6,10^ and imaging whose spatial resolution matches that of conventional MRI^11,12^.

The undesired consequence of the oscillating readout gradients during spectroscopic free induction decay are ghost spectral lines (Nyquist ghost artifacts). Ghost peaks represent “energy leakage” from the true spectral lines due to discontinuities in magnitude and phase between successive echoes^13^. The main reasons of discontinuities are imperfections in gradient hardware, eddy currents, system timing errors, susceptibility artifacts, and magnetic field inhomogeneities. Several methods for the suppression of the spectral ghost artifacts have been suggested. The simplest method is the reconstruction of the spectra from odd and even echoes separately and averaging the results^14^. The drawback of such an approach reduces the theoretically available sBW by a factor of two. Methods that maintain the full sBW combine both the odd and even echoes in spectrum reconstruction. Such data processing procedures are based on interlaced Fourier transform^15^, Fourier shift^16^, and echo shifting^13^. However, none of these methods offers entire elimination of the ghost artifact, and their effectiveness was not verified for data acquired by two or more interleaved gradient echo trains.

An alternative to the gradient echo train created by symmetrical positive and negative gradient pulses is the “flyback” gradient echo train^7–9,17^. The gradient waveform is asymmetric and combines short and strong rewind gradients with lower ones for the readout. It simplifies data processing because all echoes are acquired with the same polarity of the readout gradient. However, a high-power gradient system is needed to achieve sufficient sBW.

In this study, the flyback EPSI sequence was implemented in a preclinical MR scanner. The aim of this work is to visualize and quantify the ghost spectral lines produced by two, three and four interleaved flyback echo trains with different amplitudes of the readout gradients, and to demonstrate the potential of the flyback data acquisition in preclinical MR systems.

## Methods

### Animals

The Sprague Dawley rats weighing ~ 350 g were kept sedated by gas anesthesia (Sevofluran) during the scan. Rats were purchased from Taconic Biosciences, Lille Skensved, Denmark. Body temperature was maintained at 37 °C by warm air and breathing rate was monitored by a commercial probe based on an air pillow. The study was approved by the Ethics Committee for Animal Research in Uppsala (permit number: 5.8.18-13038/2019). All methods were performed in accordance with the relevant guidelines and regulations as well as ARRIVE (Animal Research: Reporting of In Vivo Experiments) guidelines.

### Data acquisition

**Fig. 1:**
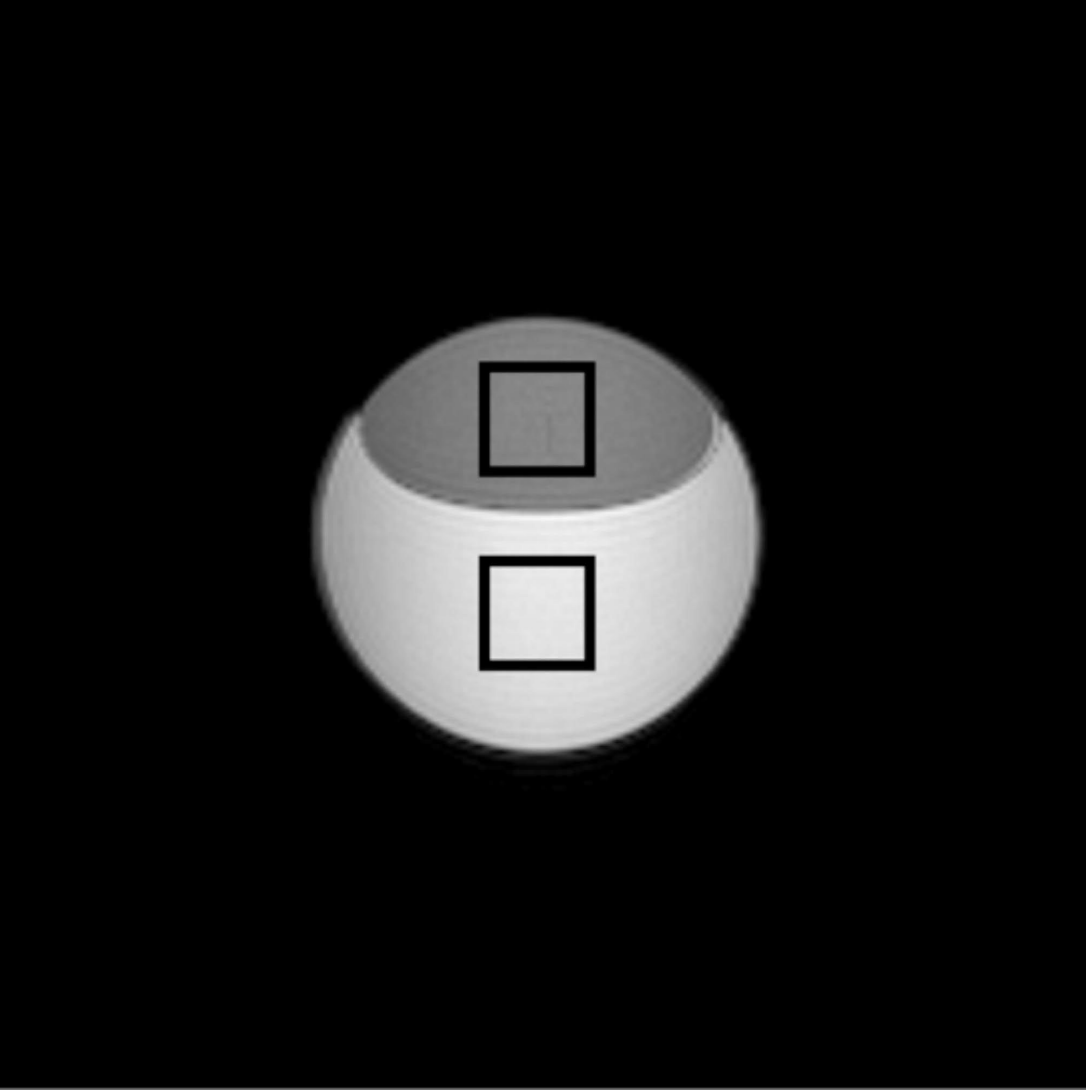
Water-fat shift artifacts free image of the phantom contained vegetable oil and water. Spectroscopic volume of interests in oil (up) and water (bottom) contain 400 voxels (51 mm^3^).

Experiments were performed on a nanoScan^®^ PET/MRI 3T preclinical animal system (Mediso Medical Imaging Systems, Budapest, Hungary). The scanner was equipped with gradients with a maximum amplitude of 550 mT/m and a maximum slew rate of 4500 T/m/s. First-order shimming was applied to improve magnetic field homogeneity before each experiment. 2D flyback EPSI sequence began with the slice selection (without water or fat suppression) followed by phase-encoding^7^. The experiments were performed with one, two, three, or four interleaved gradient echo trains with 0.15 ms ramps of trapezoidal gradient pulses^3–6^. The minimum flyback (rewind gradient) time was 0.6 ms (top time 0.3 ms and 2 × 0.15 ms ramps). We note that the amplitude and length of rewind gradient depends on the echo spacing. Ghost peaks of water and fat spectral lines were visualized with a phantom (Fig. 1) containing vegetable oil and water solution of MnCl_2_ (0.23 mM)^18^. The phantom’s T_2_ relaxation times mimics subcutaneous fat (T_2_ ~ 70 ms) and muscle water (T_2_ ~ 30 ms). The phantom experiments were performed with the maximum available FOV 80 × 80 mm, 192 phase-encoding steps, 256 sampled points per echo (matrix 192, 256), resolution in-plane 0.31 × 0.42 mm^2^, spacing between echoes ΔTE 0.8 ms (sBW 9.74 ppm), slice thickness 2 mm, 2 averages, TR/TE_1_ 220/3 ms, flip angle 30°, and with rBW 114 890, 217 014, 325 521, and 434 028 Hz. Readout bandwidths correspond to readout gradients (G_read_) of 33.73, 63.71, 95.57, and 127.42 mT/m, respectively. It was possible to perform measurement with one echo train with ΔTE 1.55 ms (sBW 5.03 ppm) and rBW 434 028 Hz. A typical PET-MRI experiment begins with PET scans that frequently take 1–2 h. In this study, images of the rats (in vivo and post-mortem) were measured with four interleaved gradient echo trains and with four echoes in each train. Twenty-seven coronal or axial slices were measured with FOV 80 × 80 mm, matrix (192, 256), resolution in-plane 0.31 × 0.42 mm^2^, slice thickness 1.3 mm, gap 0.2 mm, TR/TE_1_ 420/3 ms, ΔTE 0.62 ms (sBW 12.6 ppm), flip angle 60°, 2 averages, and rBW 169 837 Hz. The acquisition time was 10 min and 53 s. Spectra of the rat were measured with two echo trains and a total of 96 echoes (1 slice, FOV 80 × 80, matrix 192 × 256, resolution in-plane, 0.31 × 0.42, 2 averages, TR/TE_1_ 200/3, ΔTE 0.8 ms (sBW 9.74 ppm), flip angle 45°, rBW 434 028 Hz, acquisition time 2 min and 37 s).

### Data processing

Data processing software was developed in-house and is described in more detail elsewhere^10,19–21^. Briefly, the measured data (k_read_, k_phase_, k_t_) = (256, 192, k_t_) were zero-filled to the matrix (256, 256, 256). The first fast Fourier transform (FFT) was performed in the spectroscopic free induction decay direction k_t_. Spectra for each coordinate (k_read_, k_phase_) were then shifted about 1.65 ppm, i.e. by the half distance between methylene fat (-CH_2_-)n and water line^20^. This improves robustness of the (-CH_2_-)n line to spectral aliasing due to magnetic field deviations. Data processing continued by chemical shift artifact correction using a first-order phase correction in spectral domain followed by 2D FFT along k_read_ and k_phase_ dimensions^4,19^. Complex voxel spectra were then converted to magnitude spectra. Deviations of the static magnetic field ΔB from B_0_ in each voxel were computed from the position of the highest spectral line, i.e. either from water or fat (-CH_2_-)n line^19^. Voxels with highest fat line have to be recognized and the chemical shift between water and fat line 3.3 ppm must be added to the computed ΔB. The region-growing algorithm was used for this purpose^21^. We assumed that the smallest ΔB is always in the central area of the slice where the spectral line with good SNR and the position closest to the reference frequency (0 ppm) was assigned as water line and used as the starting point for region-growing algorithm. The voxel spectra were then shifted about ΔB/B_0_ along the spectral axis. Water and fat images were computed by integrating water and fat voxel spectral lines. The average magnitude spectrum per voxel was computed by the summation of the voxel spectra from the considered volume of interest (Fig. 1) and division by the number of voxels. The spectra were fitted in time domain by AMARES algorithm^22^ that is a part of the Magnetic Resonance User Interface (MRUI) software package^23^. Complex input FIDs for MRUI were computed by inverse FFT of the magnitude spectra where even and odd points of the module spectrum served as real and imaginary data for inverse FFT^24,25^.

## Results

**Fig. 2:**
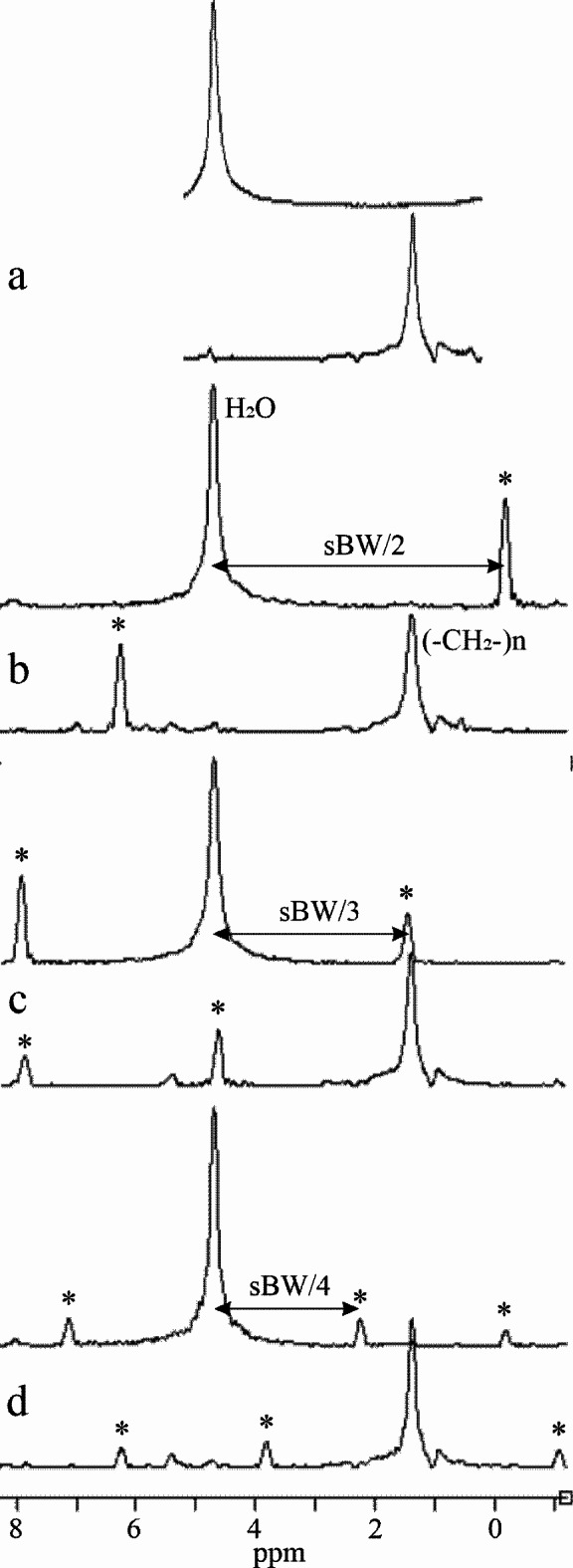
Water and vegetable oil magnitude spectra measured with 96 echoes, spectral resolution 0.1 ppm, and rBW 434 028 Hz (G_read_ 127.42 mT/m). Water line was placed at 4.7 ppm. Vegetable oil methylene (-CH_2_-)n, methyl CH_3_, and unsaturated olefinic -CH = CH- lines can be seen at 1.4, 0.9 and 5.4 ppm, respectively. Spectra were measured with (**a**) one echo train, interleaved (**b**) two, (**c**) three, and (**d**) four echo trains. The ghost peaks of water and fat (-CH_2_-)_n_ lines are marked by *.

**Fig. 3:**
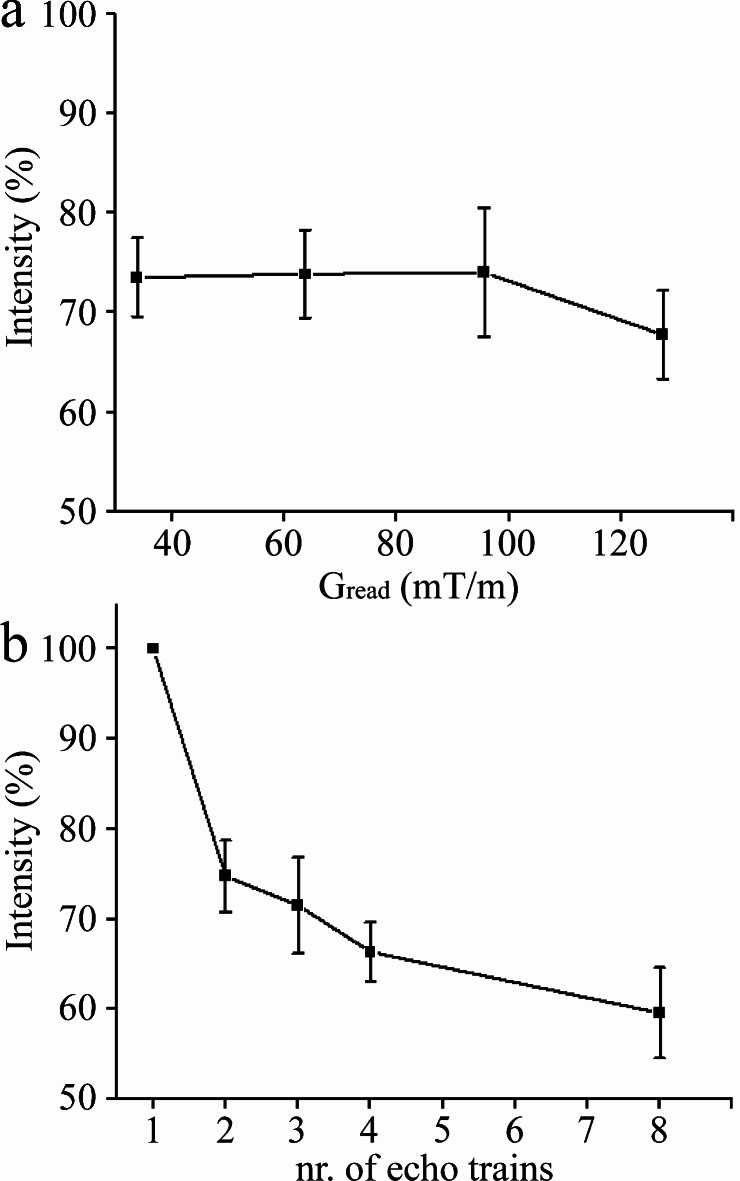
The “energy leakage” from the true water line to its ghost peaks is expressed as the intensity ratio of the true water line to the sum of true water and ghost lines. (**a**) Dependence of the intensity ratio on a readout gradient amplitude G_read_. Water spectra were acquired with four interleaved echo trains. (**b**) Dependence of the intensity ratio on the number of interleaved echo trains.

**Fig. 4:**
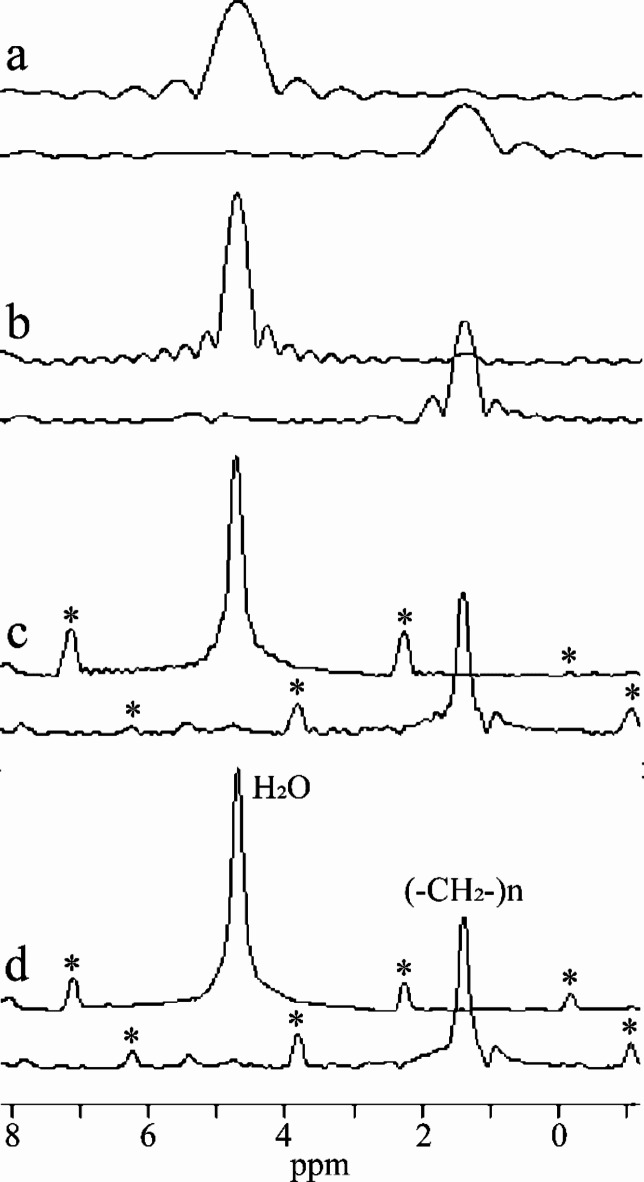
Water and vegetable oil magnitude spectra measured with four interleaved echo trains and rBW 114 890 Hz. The spectra were measured and computed with (**a**) 16, (**b**) 32, (**c**) 64 and (**d**) 96 echoes. Corresponding spectral resolutions were (**a**) 0.61, (**b**) 0.31, (**c**) 0.15, and (**d**) 0.1 ppm. The ghost peaks of water and fat line (-CH_2_-)_n_ are marked by *.

**Fig. 5:**
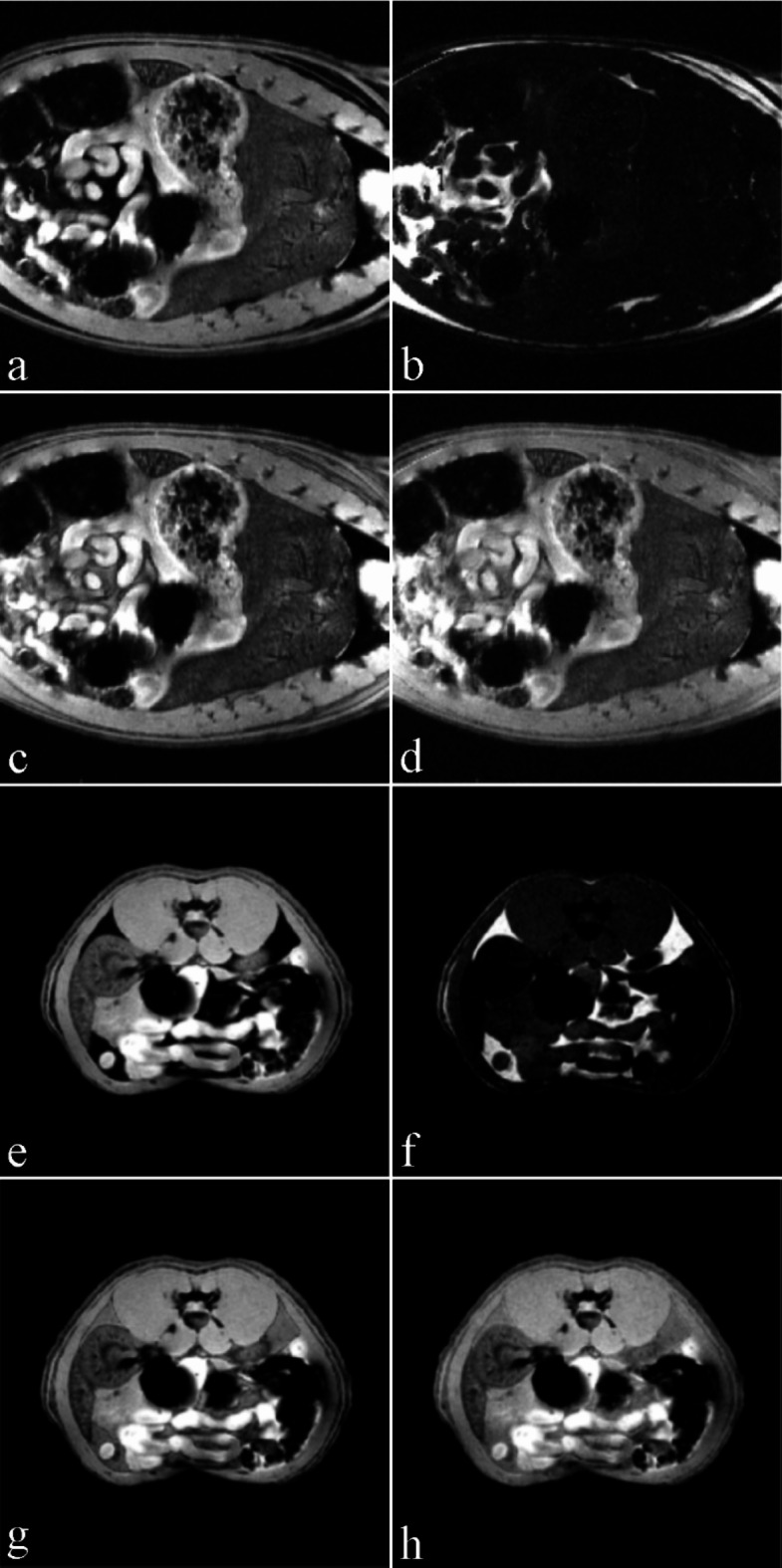
Coronal and transversal images of the dead rat. (**a,e**) water images, (**b,f**) fat images, (**c,g**) WFS artifacts free images of the highest spectral line height in the voxel. (**d,h**) WFS artifacts free images (water and fat spectral lines integrals).

**Fig. 6:**
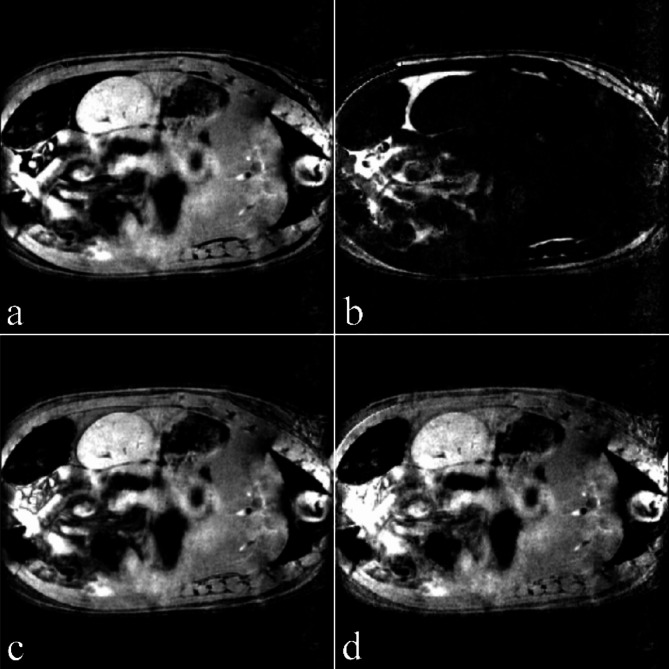
Para-coronal in vivo images of the rat. (**a**) Water image, (**b**) fat image, (**c**) WFS artifacts free image of the highest spectral line height in the voxel, (**d**) WFS artifacts free image (water and fat spectral lines integrals).

**Fig. 7:**
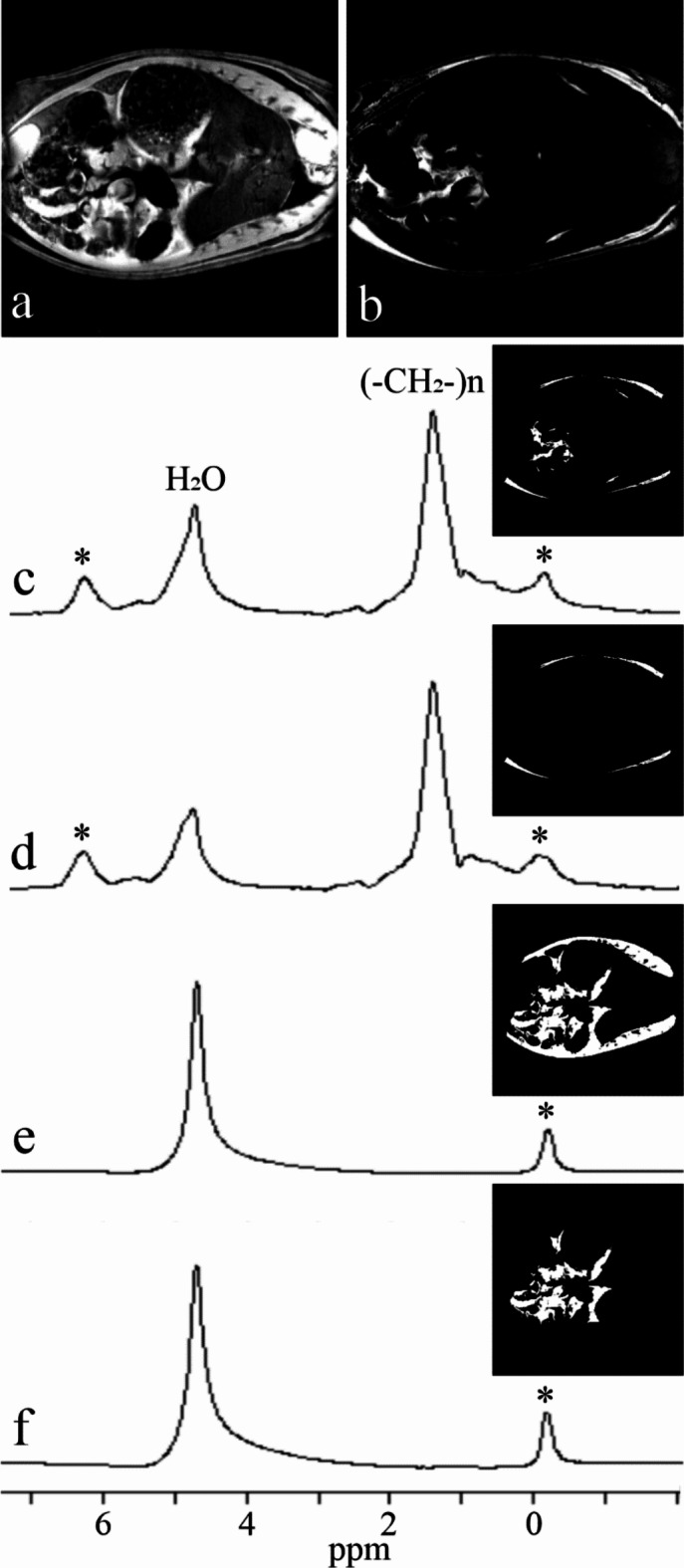
Spectra of the dead rat (spectral resolution 0.1 ppm, sBW 9.74 ppm). Volume of interests (VOIs) were made by thresholding water (**a**), and fat (**b**) images. Spectra from VOIs containing (**c**) subcutaneous and visceral fat, (**d**) subcutaneous fat, (**e**) muscles, intestines, and other abdomen tissues, and (**f**) intestines and other abdomen tissues. VOIs (white pixels) are shown on the right side of each spectrum. The ghost peaks of water and fat line (-CH_2_-)_n_ are marked by *.

The described data processing provides magnitude spectra, water, fat, and water-fat-shift (WFS) artifact-free images. Water and vegetable oil spectra were computed from the volumes of interest 51 mm^3^ (Fig. 1). The interleave of two, three, and four echo trains produce one, two, and three ghost spectral lines, respectively (Fig. 2). One echo train did not produce ghost peaks as expected (Fig. 2a). Distances between ghost peaks and main water or fat (-CH_2_-)n lines are sBW/2, sBW/3, and one or two sBW/4 for spectra acquired with two, three, and four interleaved echo trains, respectively. Figure 3 shows the “energy leakage” from the true water line to its ghost peaks expressed as the intensity ratio of the true water line to the sum of true water and ghost lines. Figure 3a illustrates the dependence of main water line intensity “leakage” on the readout gradient amplitude G_read_. The spectra were acquired with four interleaved echo trains, and total 96 echoes. The amplitudes of G_read_ were 33.73, 63.71, 95.57, and 127.42 mT/m. Figure 3b demonstrates the dependence of main water line intensity “leakage” on the number of interleaved echo trains. The experiments were performed with G_read_ 127.42 mT/m. The total number of echoes was 96. Five measurements for each G_read_ and for each number of interleaves were performed in different measurement sessions. Water and vegetable oil spectra measured with four interleaved echo trains are shown in Fig. 4. From the microimaging point of view this approach provides fairly narrow rBW (114 890 Hz) and acceptable water-fat signal separation for imaging already for 16 gradient echoes (Fig. 4a). The intensities of ghost peaks are indistinguishable from the amplitudes of truncation artifacts even for 32 echoes (Fig. 4b). Truncation artifacts are very small for 64 echoes (Fig. 4c) and totally disappear for 96 echoes (Fig. 4d). Inspired by the spectrum shown in Fig. 4a, water, fat, and WFS artifact free images of the rat were measured with four interleaved echo trains and with four echoes in each train. Post-mortem and in vivo images of the rats are shown in Figs. 5 and 6, respectively. It should be noted that in vivo images (Fig. 6) were acquired from the regions that are most influenced by motions caused by the heart, lungs, and peristaltic motions of the intestines. The examples of the rat spectra measured a few minutes after its death are shown in Fig. 7.

## Discussion

Our results have proved the feasibility of water-fat micro-imaging and spectroscopy in a preclinical MR scanner using the multislice flyback EPSI. This technique benefits from the high gradient amplitudes and slew rates available in a superconductive magnet with small bore size. High quality of water-fat images and spectra with sufficient spectral resolution were acquired in an acceptable measurement time. To our knowledge, this is the first report that demonstrates and quantifies the ghost spectral lines produced by two and more interleave gradient echo trains.

Flyback gradient echo train was introduce to EPSI by Cunningham et al. for the spectroscopy of brain metabolites^8^. The echo train was implemented in a point resolved spectroscopy (PRESS) pulse sequence. Spatial resolution and quality of the spectra were comparable to conventional proton echo-planar spectroscopic imaging (PEPSI)^14^. The advantage of the flyback echo train is reduced sensitivity to timing errors, eddy currents effects, and easier data processing due to the regular sampling of spectroscopic free induction decay^8,9^. However, flyback EPSI had not yet been used for high spatial resolution imaging in clinical scanners in spite of improvements in gradient hardware performance. The high spatial and spectral resolution of the conventional EPSI was previously used for water-fat imaging and spectroscopy of the human head^5,26^, breast^11,12,27^, pelvis^28^, calf^10^, and bone marrow^6^. The experiments were performed with one^11,12,26–28^, two^5,10^, and six^6^ interleaved symmetrical gradient echo trains. Water peak height images have been used for the detection of breast cancer lesions. The shape of the individual voxel water spectral line, its asymmetry and the formation of water line shoulder following contrast agent administration was proposed as a marker for tumor microvasculature^27,29^. The high spatial resolution permits quantification of lipids in skeletal muscles, bone marrow, liver, heart, etc. The individual voxel spectra can be summed from noncontiguous and irregularly shaped volume of interests^10^.

The experiments for ghost peaks visualization (Fig. 2) were performed with the same readout gradient for all interleaves of gradient echo trains to exclude eventual dependence of the ghost peak intensity on readout gradient amplitude. Only ghost peaks from the highest fat (-CH_2_-)n line could be recognized. Ghost peaks of other fat spectral lines were too small to be detected. One echo train does not produce ghost peaks (Fig. 2a) because ghost artifacts are created by inconsistencies between different echo trains. It’s important to note that in this study, this approach was not suitable for imaging or spectroscopy due to the main fat line aliasing on the spectral axis caused by hardware-limited narrow sBW (5.03 ppm). While one gradient echo train with symmetrical readout pulses generates one ghost peak^13,15,30^ for considered main spectral line, two interleaves of flyback echo trains are needed to produce one ghost peak. This is expected because both approaches contain two different sets of gradient echoes that always possess some inconsistencies between consecutive echoes. Since ghost peak positions depend on sBW, its value must be appropriately chosen to avoid the undesired superposition of water and fat ghost peaks with the main water and fat lines. Figure 2c illustrates such superpositions.

The “leakage” of the main water line intensity to the ghost peaks was evaluated as a function of readout gradient amplitude (Fig. 3a). From Fig. 3a, it follows that the intensity of main water spectral line is almost constant up to readout gradient amplitude 95.57 mT/m. It reveals robustness of the flyback approach to the errors caused by eddy currents^8^. The intensity of main water line decreases at highest readout gradient 127.42 mT/m, probably due to eddy currents that gradient active shielding was unable to compensate. Dependence of the main water line intensity on the number of interleaved echo trains is shown in Fig. 3b. The measurements were performed with the same amplitude of the readout gradient (127.42 mT/m) and the same total number of echoes (96). The intensity of main water line decreases with the increased number of echo train interleaves. This can be explained by the fact that each echo train interleave introduces another discontinuity in magnitude and phase of successive echoes. Relatively large standard deviations in Fig. 3 were probably caused by variations in the background gradients (magnetic field inhomogeneities) because the experiments were performed in different measurement sessions with different shimming results.

Figures 5 and 6 show water, fat, and WFS artifact free images of the rat measured with four interleaved echo trains and with four echoes in each train. This approach utilizes good water-fat spectral line separation for 16 echoes (Fig. 4a), undetectable ghost peaks, and narrow rBW. The advantage of the proposed micro-imaging approach over the conventional spin or gradient echo MRI is the fact that WFS artifacts are eliminated during data processing. Since SNR is directly proportional to (WFS)^½^ and WFS is indirectly proportional to rBW, larger WFS artifacts during acquisition can be chosen for SNR improvement without the penalty of WFS artifacts^19^. Narrow rBW can be achieved by increasing the number of interleaved echo trains. In contrast, two gradient echo interleaves are necessary for spectroscopy (Fig. 7) because the ghost peaks of water and methylene fat line are outside the interval ~(0.5; 6) ppm that is needed for lipid as well as brain metabolite spectroscopy (Fig. 2a).

This study has several limitations. The main limitation of the flyback gradient echo train sequence is decreased sampling of spectroscopic free induction decay (decreased sBW) compare to the standard EPSI with the symmetrical bipolar gradient echo train. The elimination of ghost spectral lines requires sophisticated post-processing that was not yet studied for flyback approach. A further limitation is the fact that a high-power gradient system is needed to achieve sufficient sBW (~ 9 ppm) with one or two interleaved echo trains.

## Conclusion

This work demonstrates the feasibility of echo-planar spectroscopic imaging with flyback readout gradients in preclinical MR systems. The proposed approach provides broad sBW and good water-fat signal separation for even short echo trains. Narrow rBW can be achieved by increasing the number of interleaved gradient echo trains. It improves SNR without the undesired consequence of WFS artifacts that are eliminated during data processing. Position, number, and intensity of the ghost spectral lines can be controlled by the suitable choice of sBW, number of echo train interleaves, and the number of echoes in each interleave.

## Data Availability

The datasets generated during and/or analyzed during the current study are available from the corresponding author on reasonable request.

## References

[1] Mansfield, P. Spatial mapping of the chemical shift in NMR. *Magn. Reson. Med.*** 1**, 370–386 (1984). 6571566 10.1002/mrm.1910010308

[2] Macovski, A. & Volumetric NMR imaging with time-varying gradients. *Magn. Reson. Med.*** 2**, 479–489 (1985). 3831675 10.1002/mrm.1910020105

[3] Matsui, S., Sekihara, K. & Kohno, H. Spatially resolved NMR spectroscopy using phase-modulated spin-echo trains. *J. Magn. Reson.*** 67**, 476–490 (1986).

[4] Twieg, D. B. Multiple-output chemical shift imaging (MOCSI): A practical technique for rapid spectroscopic imaging. *Magn. Reson. Med.*** 12**, 64–73 (1989). 2607962 10.1002/mrm.1910120108

[5] Ericsson, A., Weis, J., Sperber, G. O. & Hemmingsson, A. Measurements of magnetic field variations in the human brain using a 3D-FT multiple gradient echo technique. *Magn. Reson. Med.*** 33**, 171–177 (1995). 7707906 10.1002/mrm.1910330205

[6] Hilaire, L., Wehrli, F. W. & Song, H. K. High-speed spectroscopic imaging for cancellous bone marrow R_2_^*^ mapping and lipid quantification. *Magn. Reson. Imag.*** 18**, 777–786 (2000). 10.1016/s0730-725x(00)00165-x 11027870

[7] Mulkern, R. V. & Panych, L. P. Echo planar spectroscopic imaging. *Concepts Magn. Reson.*** 13**, 213–237 (2001).

[8] Cunningham, C. H. et al. Design of flyback echo-planar readout gradients for magnetic resonance spectroscopic imaging. *Magn. Reson. Med. ***54**, 1286–1289 (2005). 16187273 10.1002/mrm.20663

[9] Chen, A. P. et al. High-speed 3T MR spectroscopic imaging of prostate with flyback echo-planar encoding. *J. Magn. Reson. Imag.*** 25**, 1288–1292 (2007). 10.1002/jmri.20916 17520729

[10] Weis, J., Bruvold, M., Ortiz-Nieto, F. & Ahlström, H. High-resolution echo-planar spectroscopic imaging of the human calf. *PLOS ONE.* **9**(1), e87533 (2014). 10.1371/journal.pone.0087533 PMC3907517 24498129

[11] Du, W. et al. Breast MR imaging with high spectral and spatial resolutions: Preliminary experience. *Radiology*. **224**, 577–585 (2002). 12147859 10.1148/radiol.2242011022

[12] Medved, M., Ivancevic, M. K., Olopade, O. I., Newstead, G. M. & Karczmar, G. S. Echo-planar spectroscopic imaging (EPSI) of the water resonance structure in human breast using sensitivity encoding (SENSE). *Magn. Reson. Med.*** 63**, 1557–1563 (2010). 20512859 10.1002/mrm.22332 PMC3065325

[13] Du, W., Du, Y. P., Fan, X., Zamora, M. A. & Karczmar, G. S. Reduction of spectral ghost artifacts in high-resolution echo-planar spectroscopic imaging of water and fat resonances. *Magn. Reson. Med.*** 49**, 1113–1120 (2003). 12768590 10.1002/mrm.10485

[14] Posse, S., Tedeschi, G., Risinger, R., Ogg, R. & Le Bihan, D. High speed ^1^H spectroscopic imaging in human brain by echo planar spatial-spectral encoding. *Magn. Reson. Med.*** 33**, 34–40 (1995). 7891533 10.1002/mrm.1910330106

[15] Metzger, G. & Hu, X. Application of interlaced Fourier transform to echo-planar spectroscopic imaging. *J. Magn. Reson. ***125**, 166–170 (1997). 9245375 10.1006/jmre.1997.1114

[16] Hanson, L. G., Schaumburg, K. & Paulson, O. B. Reconstruction strategy for echo planar spectroscopy and its application to partially undersampled imaging. *Magn. Reson. Med.*** 44**, 412–417 (2000). 10975893 10.1002/1522-2594(200009)44:3<412::aid-mrm11>3.0.co;2-p

[17] Feinberg, D. A., Turner, R., Jakab, P. D. & von Kienlin, M. Echo-planar imaging with asymmetric gradient modulation and inner-volume excitation. *Magn. Reson. Med. ***13**, 162–169 (1990). 2319932 10.1002/mrm.1910130116

[18] Thangavel, K. & Saritas, E. Ü. Aqueous paramagnetic solutions for MRI phantoms at 3 T: A detailed study on relaxivities. *Turk. J. Elec Eng. Comp. Sci. ***25**, 2108–2121 (2017).

[19] Weis, J., Ericsson, A. & Hemmingsson, A. Chemical shift artifact-free microscopy: Spectroscopic microimaging of the human skin. *Magn. Reson. Med.*** 41**, 904–908 (1999). 10332872 10.1002/(sici)1522-2594(199905)41:5<904::aid-mrm8>3.0.co;2-4

[20] Weis, J. et al. Lipid content in the musculature of the lower leg: evaluation with high-resolution spectroscopic imaging. *Magn. Reson. Med. ***54**, 152–158 (2005). 15968653 10.1002/mrm.20518

[21] Weis, J., Ericsson, A. & Hemmingsson, A. Spectroscopy of the large volumes: spectroscopic imaging of total body fat. *Magn. Reson. Imag. ***19**, 1239–1243 (2001). 10.1016/s0730-725x(01)00454-4 11755735

[22] Vanhamme, L., van den Boogaart, A. & van Huffel, S. Improve method for accurate and efficient quantification of MRS data with use of prior knowledge. *J. Magn. Reson. ***129**, 35–43 (1997). 9405214 10.1006/jmre.1997.1244

[23] Naressi, A. et al. Java-based graphical user interface for the MRUI quantitation package. *MAGMA*. **12**, 141–152 (2001). 11390270 10.1007/BF02668096

[24] Weis, J., Johansson, L., Courivaud, F., Karlsson, F. A. & Ahlström, H. Quantification of intramyocellular lipids in obese subjects using spectroscopic imaging with high spatial resolution. *Magn. Reson. Med. ***57**, 22–28 (2007). 17152088 10.1002/mrm.21085

[25] Sorensen, H. V., Jones, D. L., Heideman, M. T. & Burrus, C. S. Real-valued fast Fourier transform algorithms. *IEEE Trans. Acoust. Speech Sig Process. ***35**, 849–863 (1987).

[26] Du, W., Karczmar, G. S., Uftring, S. J. & Du, Y. P. Anatomical and functional brain imaging using high-resolution echo-planar spectroscopic imaging at 1.5 Tesla. *NMR Biomed.*** 18**, 235–241 (2005). 15759296 10.1002/nbm.952

[27] Medved, M. et al. The effect of varying spectral resolution on the quality of high spectral and spatial resolution magnetic resonance images of the breast. *J. Magn. Reson. Imag. ***18**, 442–448 (2003). 10.1002/jmri.10378 14508781

[28] Sarkar, S., Heberlein, K., Metzger, G. J., Zhang, X. & Hu, X. Application of high-resolution echoplanar spectroscopic imaging for structural imaging. *J. Magn. Reson. Imag.* **10**, 1–7 (1999). 10.1002/(sici)1522-2586(199907)10:1<1::aid-jmri1>3.0.co;2-c 10398971

[29] Foxley, S. et al. Sensitivity to tumor microvasculature without contrast agents in high spectral and spatial resolution MR images. *Magn. Reson. Med. ***61**, 291–298 (2009) 10.1002/mrm.21801 PMC3370261 19165878

[30] Ebel, A., Maudsley, A. A., Weiner, M. W. & Schuff, N. Achieving sufficient spectral bandwidth for volumetric ^1^H echo-planar spectroscopic imaging at 4 Tesla. *Magn. Reson. Med.*** 54**, 697–701 (2005). 16086316 10.1002/mrm.20593 PMC1851680

